# 

**Published:** 1885-04-20

**Authors:** 


### EDITORIALS AND MISCELLANEOUS.

☞ **Notice.**--- Subscribers in arrears are requested to remit their dues at once. Please take due notice and govern yourselves accordingly.

### EDITORIAL NOTICES.

SEE advertisement of Peacock Chemical Co. in this issue.

THE following are the officers of the International Medical Congress of 1887:

Dr. Austin Flint, Sr., of New York, President; Dr. Alfred Stille, of Philadelphia, Dr. Henry I. Bowditch, of Boston, and Dr. R. P. Howard, of Montreal, Canada, Vice-Presidents; Dr. John S. Billings, of the Army, Secretary General; Dr. J. M. Browne, of the Navy, Treasurer: and Dr. I. Minis Hays, of Philadelphia. Dr. A. Jacobi, of New York, Dr. Christopher Johnston, of Baltimore, and Dr. S. C. Busey, of Washington, members of the Executive Committee.

THE WORLD'S EXPOSITION.—Only a few weeks remain during which the people can see this grand World's Exposition of the Progress and Development of the vast resources of our country, the marvelously complete exhibit of Mexico, together with the endless collections from all parts of the globe, the whole made more delightful by the sweet music of the Mexican Military Band, with its seventy-three trained musicians, not excelled by any band ever organized. The Exposition closes May 31st, 1885.

PEPTOGENIC MILK POWDER.—We believe this preparation to be excellent for the purpose named. It is prepared by that large and widely known establishment of New York and London known as Fairchild, Bros. & Foster.

Their polite and intelligent agent called on us and exhibited the practical workings of the preparation, presenting us with a sample of humanized mother's milk, the caseine of the cow's milk being modified and predigested and other components so supplied as to constitute a combination physiologically identical with human milk. See the advertisement of this house in our Journal.

ACCOMPANYING the card of Dr. Gaston requesting reports of surgical operations upon the gall-bladder and ducts, there appears in the Medical Press and Circular of London, England, the following unique editorial comment:

"For our correspondent's information, we might mention that, in this country, when an author is engaged in any literary work he usually looks up references for himself, not considering it altogether reasonable to ask strangers to undertake the labor for him. If, however, Dr. Gaston should be fortunate enough to enlist the gratuitous services of any of our readers, it will afford us pleasure to congratulate him on an unexpected success.—ED."

Whether this is intended as a caution to American medical men who may look to English authors for facts connected with their experience, or as a criticism upon the conservative element of the British profession, it most assuredly is not indorsed by the practice of the English physicians of greatest prominence, nor by those of highest distinction in other countries.

We are happy to know that strangers become friends in the interchange of medical and surgical information between the members of this brotherhood. Dr. Gaston has received ample evidence of professional courtesy from English and other European colleagues in the valuable data furnished on the surgery of the gall-bladder, and authorizes us to express his appreciation of their generosity, the opinion of the Press and Circular to the contrary, notwithstanding.

### *RECEIPTED.*

1885.—Drs. L. M. Johnson to July, J. R. Wilson, R. C. Wyly, J. Dillworth, Jeff. Willcox.

1884.—Drs W. H. Peek, G. M. McMillan, J. A. Holloway to July, James Alston, E. K. Miller, Thos. A. Lehman, R. Middlebrooks, Evan T Jones, M. M Denham, Joseph Jolly, Morrison Thompson, James Cater, John McMullan, M. P. Deadwyler,

